# Experiences and lessons learned from the Nigeria polio program

**DOI:** 10.11604/pamj.supp.2023.45.2.41049

**Published:** 2023-07-13

**Authors:** Faisal Shuaib, Omotayo Bolu, Usman Adamu, Adeyelu Asekun

**Affiliations:** 1 National Primary Healthcare Development Agency, Abuja, Nigeria,; 2 Centers for Disease Control and Prevention, Atlanta, Georgia, United States,; 3 National Emergency Operation Centre, Abuja, Nigeria

**Keywords:** Wild poliovirus, global polio eradication initiative, oral polio vaccine, Nigeria

## Editorial

Since 2016, Nigeria has remained free from the indigenous wild poliovirus (WPV), a success that led to the WPV certification of the African region in August 2020. Despite this great achievement, in 2021, Nigeria recorded the largest number of circulating variant poliovirus type 2 (cVPV2) outbreaks [1] since the switch from the trivalent oral polio vaccine (tOPV) to the bivalent oral polio vaccine. Thirty-one states reported confirmed cVPV2s and the country-initiated robust response to the outbreaks using the novel oral polio type 2 (nOPV2) vaccine, a more genetically stable version of the monovalent oral polio vaccine type 2 (mOPV2) [2]. By December 2021, nOPV2 had been deployed across all thirty-six states and the Federal Capital Territory (FCT). However, despite the robust response to the cVPV2 outbreaks, transmission persisted across several states in the country [3], due to several factors including difficulty in accessing key reservoirs and the impact of the COVID-19 pandemic on the surveillance system [4]. In 2022, the country continued its efforts against cVPV2 transmission and conducted several outbreak responses in close collaboration with the states to ensure optimal implementation of strategies and quality interventions for improved uptake of polio vaccines especially in insecure and underserved communities.

In 2023, the National Primary Health Care Development Agency (NPHCDA) jointly with Global Polio Eradication Initiative Partners (GPEI) and other stakeholders have made it a key priority to end all forms of polioviruses transmission around the world by rebuilding program resilience and focusing on the most consequential geographies and locations at the highest risk of continued transmission and spread of the virus. These consequential geographies have been identified in Nigeria, mostly in the northern part of the country, hyper-localized to specific local government areas (LGAs) with the highest number of unimmunized children. Nigeria implemented the co-administration of inactivated polio vaccine type 2 (IPV2) with nOPV2 to boost population immunity and permanently halt the cVPV2 outbreaks (WHO, 2021) and this is a recommendation the Strategic Advisory Group of Experts on Immunization endorsed in 2023 [5].

Several lessons have been well documented over the years that tell a story of polio in Nigeria and the efforts the country has made in the fight against this life-threatening and debilitating disease. These lessons can be useful for future public health interventions and can be leveraged by Nigeria in response to future outbreaks and by other countries as they develop strategies to combat polio and other vaccine-preventable diseases. This special issue of the Pan African Medical Journal documents the experiences and lessons learned from the Nigeria polio program. The papers, written by those who were at the forefront of the fight against polio in Nigeria, cover key topics from surveillance, epidemiology of polio in Nigeria, studies to boost planning and implementation of innovative strategies for outbreak response, seroprevalence study to understand the immunity gaps after the switch, deployment of the nOPV2 vaccine, and adoption of technology, and innovative strategies to mitigate the impact of COVID on polio. Embedded within the narrative of these articles are nuggets that will enhance public health programs within and outside the African Region and will be of great value to the global fight to eradicate all types of polioviruses and make polio history.

**Disclaimer:** the findings and conclusions in this report are those of the authors and do not necessarily represent the official position of the U.S. Centers for Disease Control and Prevention.
